# Ascites-derived IL-6 and IL-10 synergistically expand CD14^+^HLA-DR^-/low^ myeloid-derived suppressor cells in ovarian cancer patients

**DOI:** 10.18632/oncotarget.20164

**Published:** 2017-08-10

**Authors:** Liangliang Wu, Zhaoyang Deng, Yaojun Peng, Lu Han, Jing Liu, Linxiong Wang, Bohua Li, Jian Zhao, Shunchang Jiao, Huafeng Wei

**Affiliations:** ^1^ Key Lab of Cancer Center, General Hospital of Chinese PLA & Beijing Key Laboratory of Cell Engineering & Antibody, Beijing, China; ^2^ Surgical Division, General Hospital of Chinese PLA, Beijing, China; ^3^ Department of Internal Oncology, General Hospital of Chinese PLA, Beijing, China; ^4^ Shanghai Key Laboratory for Molecular Imaging, Shanghai University of Medicine & Health Sciences

**Keywords:** MDSC, ovarian cancer, IL-6, IL-10, STAT3

## Abstract

Myeloid-derived suppressor cells (MDSC) play a key immunosuppressive role in various types of cancer, including ovarian cancer (OC). In this study, we characterized CD14^+^HLA-DR^–/lo^ MDSC with a typical monocytic phenotype (M-MDSC) in the peripheral blood (PB) and ascites from OC patients. Compared to healthy donors, OC patients had a significantly increased abundance of M-MDSC in both PB and ascites; importantly, their abundance in both compartments was inversely associated with the prognosis where OC patients with higher level of M-MDSC having a shorter relapse-free survival. Intriguingly, we demonstrated that M-MDSC could be readily induced by ascitic fluids (AF) from OC patients, which was predominantly dependent on IL-6, IL-10 and STAT3 activation as neutralization of IL-6 and/or IL-10 or inhibition of STAT3 abrogated MDSC's expansion while recombinant IL-6 and IL-10 recapitulated the expansive effect of AF; furthermore, predominantly elevated levels of IL-6 and IL-10 has been noted in the AF which was positively correlated with the abundance of M-MDSC as well as poor prognosis of OC patients. As expected, we observed that AF-driven STAT3 activation upregulated the expression of arginase (ARG1) and inducible nitric oxide synthase (iNOS) in induced M-MDSC through which these MDSC executed the immunosuppressive activity. Taken together, these results demonstrate that abundant M-MDSC are present in both periphery and ascites of OC patients whose accumulation and suppressive activity is critically attributable to ascites-derived IL-6 and IL-10 and their downstream STAT3 signal, thus providing a potentially novel therapeutic option by locally targeting MDSC to improve antitumor efficacy.

## INTRODUCTION

OC (OC) is the most deadly gynecologic malignancy with over 70% of patients having advanced disease at the time of diagnosis. Surgical debulking in combination with platinum-based adjuvant chemotherapy remains first-line therapy to which about 80% of patients will initially respond well; however, most of these patients eventually succumb to their disease due to the outgrowth and spreading of chemoresistant cancer cells to serous membranes throughout the peritoneal cavity [1, 2]. During this metastatic process, malignant ascites is formed by a malignant effusion building up in the peritoneal cavity at advanced cancer stages, as a carrier, greatly facilitates the passive dissemination of cells shed from the primary tumor cells, and is therefore an essential determinant of metastatic dissemination [3, 4]. This malignant ascites is rich in tumor-promoting soluble factors, cancer cells and immune cells; though its role in fostering OC metastasis has been subject of intensive investigation, the contribution of its host-derived cellular constituents remains poorly understood [2, 5].

Accumulating evidence demonstrates that accumulation of pathologically activated immature myeloid-derived suppressor cells (MDSC) with potent immune-suppressive activity is one of the major immunological hallmarks of cancer progression and metastasis [6]. MDSC represent a heterogeneous population of immature myeloid cells at different stages of differentiation, including immature precursors of macrophages, granulocytes, and dendritic cells (DC) [7, 8]. In mice, MDSC were historically defined as cells expressing both Gr-1 and CD11b markers [9]. It is now established that MDSC consist of two major groups of cells with mononuclear MDSCs (M-MDSC) and polymorphonuclear MDSCs (PMN-MDSC) defined as CD11b^+^Ly6C^high^Ly6G^−^ cells and CD11b^+^Ly6C^low^Ly6G^+^ cells respectively [9]; accordingly, human equivalent to M-MDSC were defined as CD33^+^CD14^+^HLA-DR^−/low^CD15^−^cells and PMN-MDSCs as CD33^+^CD14^−^CD15^+^ or CD33^+^CD14^−^CD66b^+^ cells [10]. As an important player contributing to the immunosuppressive tumor microenvironment, MDSC's abundance was frequently reported to be markedly increased in the peripheral blood (PB) and tumor tissues from cancer patients and correlated with metastatic tumor burden, clinical stage and/or prognosis of cancer patients in many cancer types [7, 8, 11], including OC [12, 13]. Regarding OC, research group led by Freedman previously identified a subpopulation of IL-10-producing CD14^+^HLA-DR^−^ monocytes with suppressive activity against T-cell effector function and proliferation in malignant ascites from OC patients which presented the features of M-MDSC described currently [14]; furthermore, Natasa et al. reported the presence of CD11b^+^CD14^+^CD33^+^ MDSC in OC patients which was attractive into ascites by PEG2-controlled CXCL12/CXCR4 chemotactic axis [12]; recently, Cui et al. demonstrated the inverse relationship between intratumoral CD45^-^CD33^+^ MDSC content and overall survival of patients with OC [13], indicating the clinical relevance of MDSC in OC. In spite of these pioneering studies, detailed characterization of MDSC phenotype, function, and generation in the PB and/or ascites from OC patients remains to be needed.

In our study, we have defined the frequency, phenotype, generation and function of CD14^+^HLA-DR^–/lo^ MDSC in the PB and ascites isolated from OC patients. We show that these MDSC are abundantly enriched in the PB and in particular, in the ascites; they express the surface markers typical of M-MDSC, and are able to suppress autologous T-cell proliferation and effector cytokine production; more importantly, their abundance in both PB and ascites is inversely correlated with the prognosis of OC patients; We further demonstrate that these MDSC can be induced by ascitic fluids (AF) where high levels of IL-6 and IL-10 play an essential role via activation of downstream STAT3 signaling pathway. We further show that induced CD14^+^HLA-DR^–/lo^ MDSC execute the immunosuppressive function via ARG1 and iNOS upregulated by AF-driven STAT3 activation.

## RESULTS

### Increased CD14^+^HLA-DR^-/low^ MDSC in the PB and ascites from OC patients

**Figure 1:**
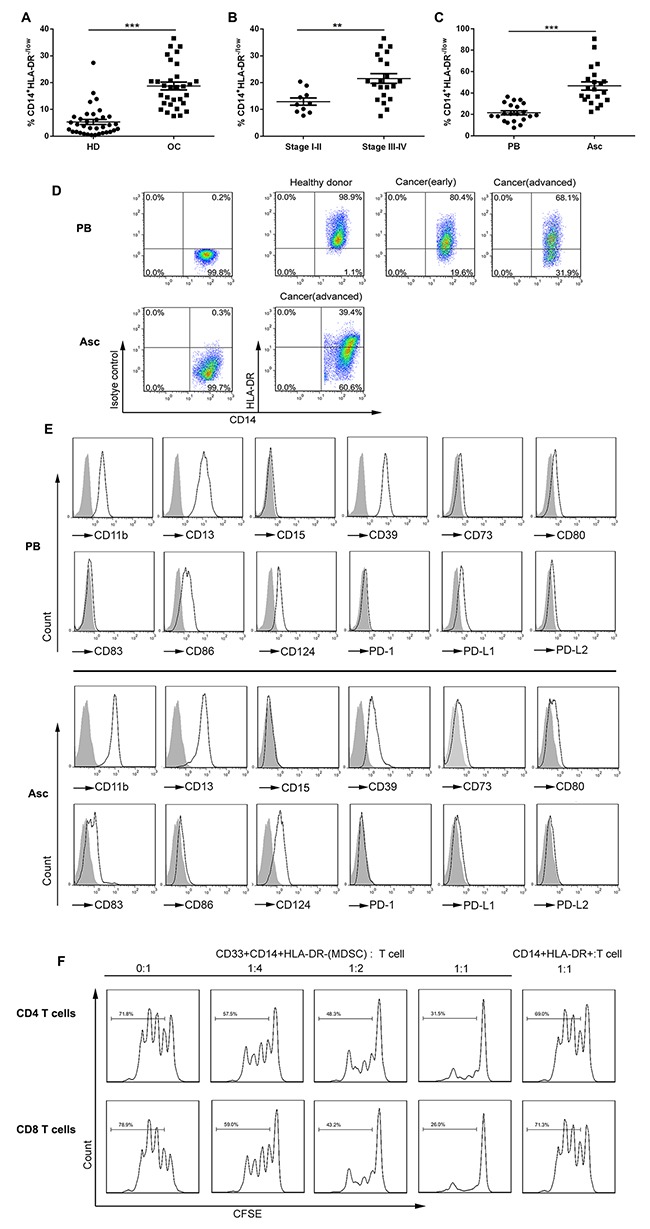
The distribution, phenotypes and suppressive function of CD14^+^HLA-DR^–/lo^ MDSC in the PB and ascites from OC patients **(A)** Relative abundance of circulating CD14^+^HLA-DR^–/lo^ MDSC (with respect to CD33^+^ cells) in OC patients (n = 31) versus healthy donors (HD; n = 35). The mean CD33^+^ cells from these populations were not statistically different. **(B)** Relative abundance of circulating CD14^+^HLA-DR^–/lo^ MDSC in OC patients with early (stage I/II; n = 10) versus advanced (stage III/IV; n = 21) disease. **(C)** Relative abundance of CD14^+^HLA-DR^–/lo^ MDSC in the PB versus accompanying ascites from the same OC patients (n = 21). **(D)** Representative dotplots of CD14^+^HLA-DR^–/lo^ MDSC in the PB and/or accompanying ascites from HD or OC patients with early or advanced disease. **(E)** Representative histograms of MDSC surface markers on CD14^+^HLA-DR^–/lo^ MDSC in the PB or accompanying ascites with isotype controls as shaded areas. **(F)** The immune suppressive activity of circulating CD14^+^HLA-DR^–/lo^ MDSC from OC patients on autologous T cell proliferation at the varying T cell/MDSC ratios with CD14^+^HLA-DR^+^ cells from the same donors as control. **p < 0.01, ***p < 0.001, unpaired student *t* test for A and B or paired student *t* test for C.

We determined the frequency of CD14^+^HLA-DR^–/low^ cells in the PB of OC patients before treatment and found that there was a larger accumulation of these potentially suppressive cells in OC patients compared to healthy donors. Though mean CD14^+^ monocytes in the PB leukocytes show no difference (Supplementary Figure 1A), mean CD14^+^HLA-DR^–/low^ cells in total CD33^+^ myeloid cells in OC patients was 18.7% ± 8.3% in comparison with 5.5% ± 5.3% in healthy donors (Figure 1A). Consistent with previous studies, we observed that the abundance of CD14^+^HLA-DR^–/low^ cells was correlated with the cancer stage where a significant increase was seen in the percentage of circulating CD14^+^HLA-DR^–/low^ cells among advanced stage (stage III/IV) OC patients (21.5% ± 8.3%) as compared with early stage (stage I/II) OC patients (12.9% ± 4.3%) (Figure 1B). No correlation was noted between the percentage of circulating CD14^+^HLA-DR^–/low^ cells and tumor grade or cancer subtype (Supplementary Figure 1B and 1C). When we analyzed CD14^+^HLA-DR^–/low^ cells in the accompanying ascites, we noted the significantly higher percentage of these cells in the ascites (46.6% ± 17.9%) as compared to that in the PB (21.5% ± 8.3%) and also an increase in the percentage of CD14^+^ monocytes (Supplementary Figure 1D). The representative dotplots for CD14^+^HLA-DR^–/low^ cells were shown in Figure 1D.

Further phenotypic analysis of surface markers showed that CD14^+^HLA-DR^–/low^ cells represented the features of M-MDSC; these cells from both PB and ascites expressed the myeloid markers CD11b, CD13, and CD39 as well as CD124, a marker of M-MDSC previously described (Figure 1E); however, CD14^+^HLA-DR^–/low^ cells from these two compartment exhibited both similarity and distinction in the expression of cosignaling (costimulatory and coinhibitory) molecules in that both had the low levels of CD80 and PD-L1 expression, but intermediate levels of CD86 or CD83 expression on circulating or ascitic cells respectively. Distinguishable from tumor-associated macrophages (TAM), we did not see expression of CD68, CD204 and CD206 expression on these cells (Supplementary Figure 1E).

We next evaluated the immunosuppressive activity of these cells by T cell/MDSC coculture assay. CD14^+^HLA-DR^–/low^ cells as well as control CD14^+^HLA-DR^+^ cells were freshly isolated from the PB of OC patients by flow cytometry using the gating strategy described in Supplementary Figure 1F and then were cocultured with CFSE-labeled autologous CD4^+^ and CD8^+^ T cells at the varying ratios; as shown in Figure 1F, CD14^+^HLA-DR^–/low^ cells were found to suppress autologous CD4^+^ and CD8^+^ T cell proliferation *in vitro* at even 4:1 T cell/MDSC ratio while CD14^+^HLA-DR^+^ cells from the same patients did not, validating the identity of MDSC for CD14^+^HLA-DR^–/low^ cells from OC at the functional level. Sorted CD14^+^HLA-DR^–/low^ cells sorted from the accompanying ascites of OC patients exhibited a similar immunosuppressive activity on autologous T cells in coculture assays (data not shown).

### Significantly elevated levels of IL-6 and IL-10 are associated with the abundance of CD14^+^HLA-DR^-/low^ MDSC in the AF

**Figure 2:**
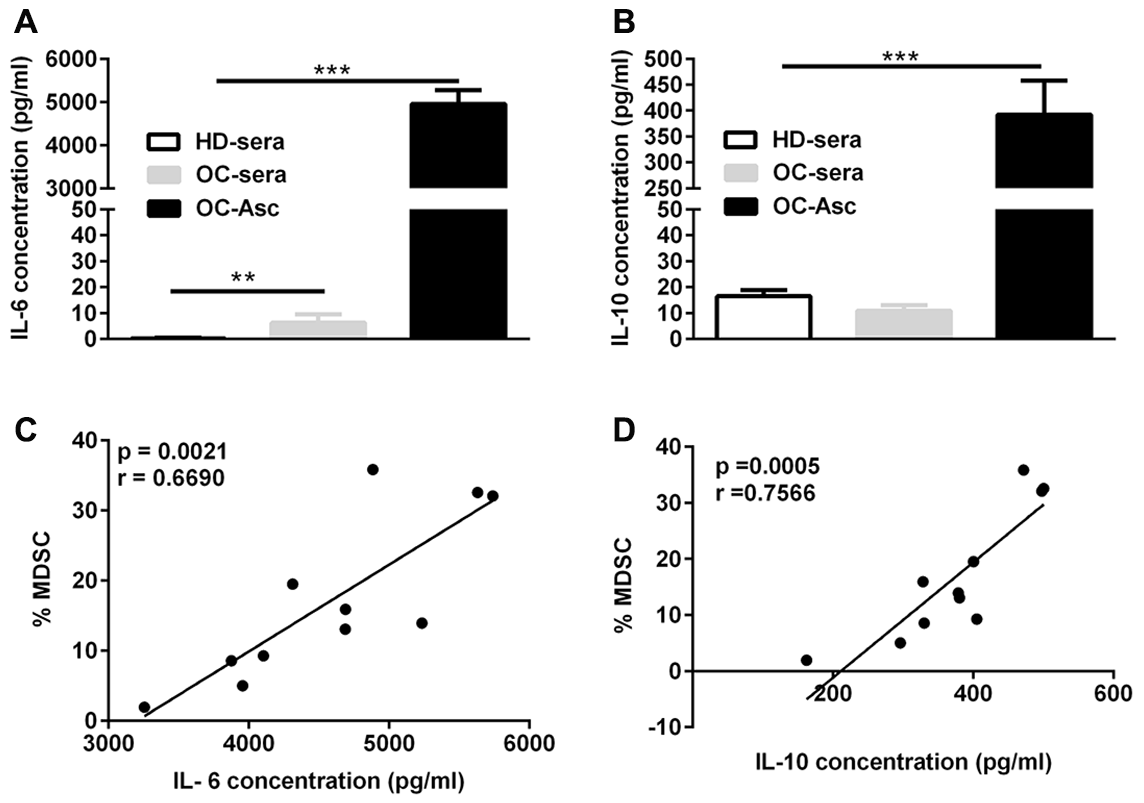
The correlation between IL-6 and IL-10 levels and the abundance of CD14^+^HLA-DR^-/low^ MDSC in the ascites **(A)** IL-6 concentration in the sera and/or accompanying ascites from HD (n = 21) or OC (n = 11) patients. **(B)** IL-10 concentration in the sera and/or accompanying ascites from HD or OC patients. **(C)** The correlation between the abundance of CD14^+^HLA-DR^–/lo^ MDSC and IL-6 in ascites from OC patients (n = 11). **(D)** The correlation between the abundance of CD14^+^HLA-DR^–/lo^ MDSC and IL-10 in ascites from OC patients (n = 11). ***p < 0.001, one-way ANOVA followed by Tukey's multiple comparisons test for A and B or Pearson test for C and D.

The data described above demonstrated a predominant increased CD14^+^HLA-DR^–/low^ cells in the accompanying ascites in comparison with the PB of OC patients; as previous studies have shown the presence of multiple inflammatory cytokines in the AF from OC patients and several inflammatory cytokines have been defined to be able to promote the expansion and accumulation of MDSC, we examined the levels of multiple inflammatory cytokines in the paired PB sera and AF from OC patients by cytokine array and then determined whether their levels were associated with the abundance of CD14^+^HLA-DR^–/low^ MDSC. Cytokine analysis demonstrated a marked elevation of IL-6 and IL-10 in the AF from OC patients as compared to the accompany sera as well as sera from healthy donor (Figure 2A and 2B); no difference in the levels of other 10 cytokines (IL-1β, IL-2, IL-4, IL-5, IL-9, IL-22, IL-13, IL-17A, IFN-γ, TNF-α) were noted (Supplementary Figure 2). Further correlation analysis confirmed a significant correlation of CD14^+^HLA-DR^–/low^ MDSC with the concentration of both IL-6 (p = 0.0021; correlation coefficient r = 0.669; Figure 2C) and IL-10 (p = 0.0005; correlation coefficient r = 0.7566; Figure 2D). None of the other cytokines tested was significantly associated with the abundance of CD14^+^HLA-DR^–/low^ MDSC (Supplementary Table 3). We also observed significant correlation between IL-6 and IL-10 (p = 0.001) while other correlations were not detectable (Supplementary Table 4).

### Association of relapse-free survival with the levels of CD14^+^HLA-DR^-/low^ MDSC, IL-6 and IL-10

**Figure 3:**
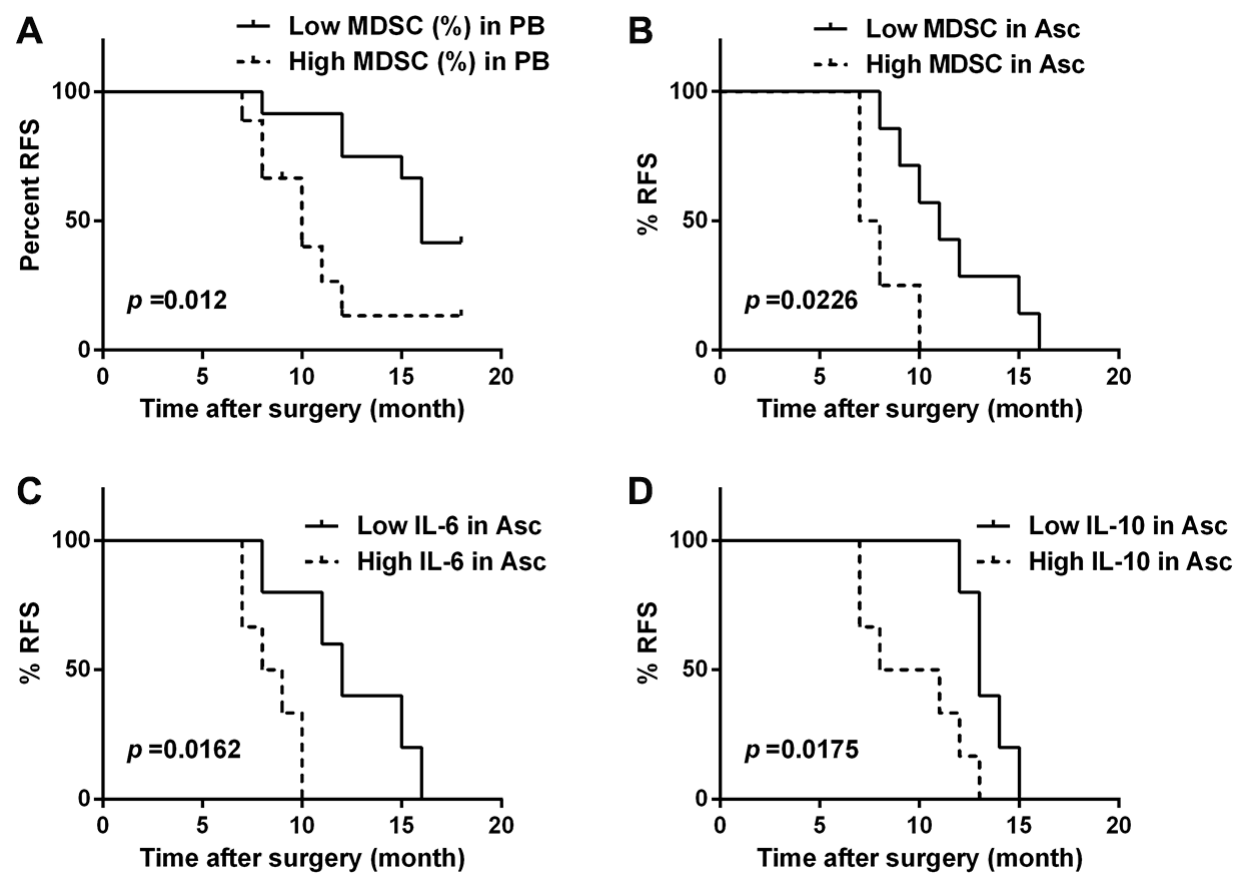
The correlation between relapse-free survival (RFS) and the abundance of CD14^+^HLA-DR^-/low^ MDSC and the levels of IL-6 and IL-10 in the OC patients **(A)** Kaplan-Meier plots showing the correlation between RFS and high or low levels (median as cutoff) of CD14^+^HLA-DR^–/lo^ MDSC in the PB **(A)** or accompanying ascites **(B)**, and IL-6 **(C)** or IL-10 **(D)** concentration in the accompanying ascites in the OC patients (n = 21 for PB and n = 11 for ascites) with p-Values determined by Mantel–Cox log-rank test.

We further analyzed potential correlations between the abundance of CD14^+^HLA-DR^–/low^ MDSC in the PB and ascites or levels of IL-6 and IL-10 in the AF and clinical progression of OC patients. All patients with a postsurgery period of at least 6 months (range 7-18 months) were included in our study (n = 21 for PB and n = 11 for ascites). For each parameter, patients were grouped as “high” or “low” using the respective median as a cutoff point. These datasets were analyzed for association with relapse-free survival (RFS). As expected, the frequency of CD14^+^HLA-DR^–/low^ MDSC in both PB and ascites was inversely associated with RFS (log-rank test, p = 0.0215 and 0.0226; Figure 3A and Figure 3B); furthermore, we also found an inverse correlation between RFS and the levels of IL-6 and IL-10 in the AF (log-rank test, p = 0.0162 and 0.0175; Figure 3C and Figure 3D). A similar negative association was also observed between RFS and tumor stage, but not histological grading (data not shown).

### Functional CD14^+^HLA-DR^-/low^ MDSC can be significantly expanded by the AF from OC patients

**Figure 4:**
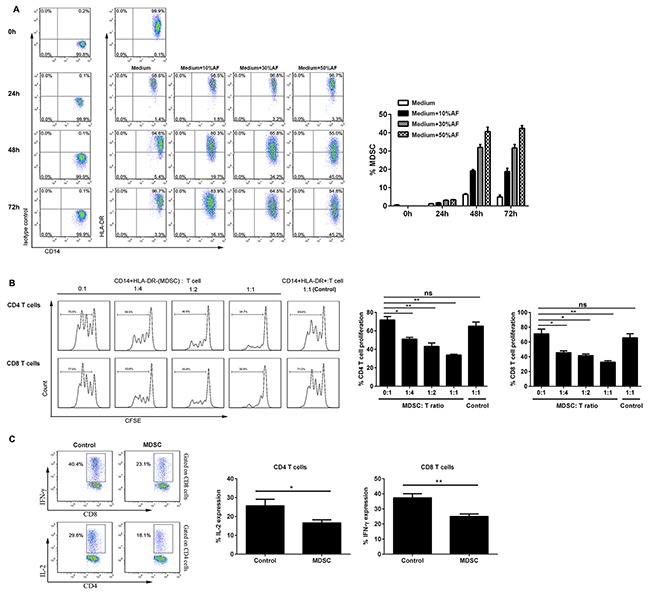
AF-driven expansion of functional CD14^+^HLA-DR^-/low^ MDSC **(A)** PBMC from HD (n = 3) were cultured in the presence of varying concentrations (10, 30, 50% v/v) of AF from OC patients (n = 4) for up to 72 hours and then analyzed for the abundance of CD14^+^HLA-DR^-/low^ MDSC by flow cytometry. The representative dotplots were shown in left panel and the statistics were shown in right graph. **(B)** Both CD14^+^HLA-DR^–/lo^ MDSC and CD14^+^HLA-DR^+^ control cells from AF-treated PBMC (30% v/v, 48 hours) were sorted by flow cytometry and then assayed immune suppressive activity on autologous T cell proliferation at the varying T cell/MDSC ratios. The representative dotplots were shown in left panel and the statistics were shown in right graph. **(C)** CD14^+^HLA-DR^–/lo^ MDSC were also assayed immune suppressive activity by evaluating IL-2 and IFN-γ production in autologous CD4^+^ and CD8^+^ T cells by intracellular staining. The data are expressed as mean ± SEM of 4 biological replicates and representative of three independent experiments. *p < 0.05, **p < 0.01, paired student *t* test for B or one-way ANOVA followed by Tukey's multiple comparisons test for C.

To define the origin of markedly increased CD14^+^HLA-DR^–/low^ MDSC in the ascites as compared to the PB from OC patients, we cultivated the freshly isolated PBMC from healthy donors in the presence of varying concentrations of AF from OC patients for up to 72 hours and then analyzed the abundance of CD14^+^HLA-DR^–/low^ MDSC by flow cytometry; as shown in Figure 4A, addition of AF from OC patients significantly potentiated the expansion of CD14^+^HLA-DR^–/low^ cells in a dose-dependent manner within 72 hours with 48 hours being the optimal time. The representative dotplots for MDSC expansion were also shown in Figure 4A.

We next examined the functionality of AF-expanded CD14^+^HLA-DR^–/low^ cells by T cell/MDSC coculture assay; as shown in Figure 4B, AF-expanded CD14^+^HLA-DR^–/low^ cells were found to suppress the proliferation of autologous CD4^+^ and CD8^+^ T cells *in vitro* as similar to that of freshly isolated circulating CD14^+^HLA-DR^–/low^ MDSC; moreover, they were also able to inhibit the production of effector cytokine IL-2 and IFN-γ of autologous CD4^+^ and CD8^+^ T cells (Figure 4C), confirming the identity of MDSC for AF-expanded CD14^+^HLA-DR^–/low^ cells at the functional levels.

### Both IL-6 and IL-10 critically contributed to AF-driven expansion of CD14^+^HLA-DR^-/low^ MDSC in a STAT3-dependent manner

**Figure 5:**
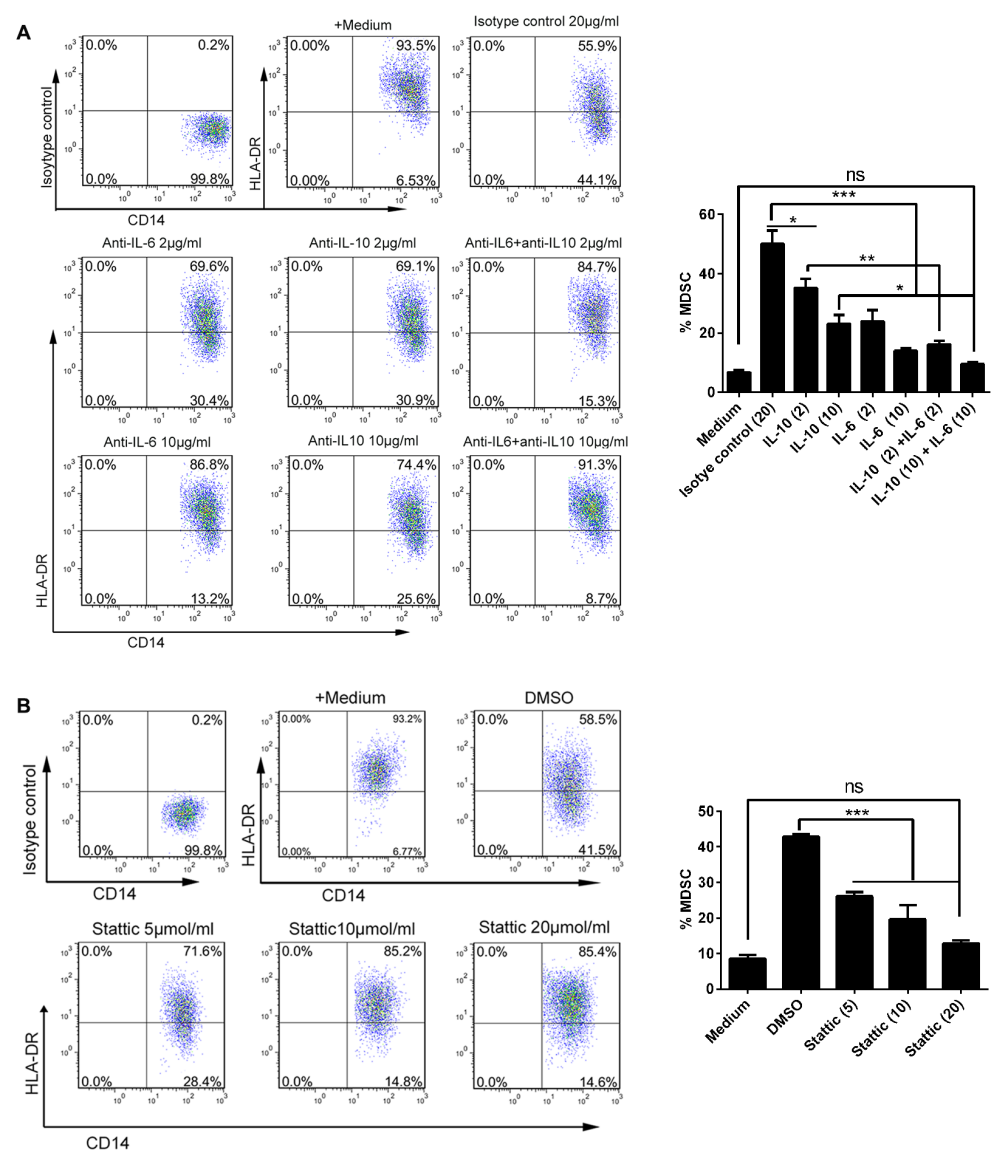
AF-driven expansion of CD14^+^HLA-DR^-/low^ MDSC was dependent on IL-6/IL10-STAT3 signal pathway PBMC from HD (n = 3) were treated with the AF (50% v/v) from OC patients (n=4) in the presence of neutralizing antibodies against IL-6 and/or IL-10 **(A)** or STAT3 inhibitor stattic **(B)** for 48 hours and then analyzed for the abundance of CD14^+^HLA-DR^-/low^ MDSC by flow cytometry. Addition of isotype antibody or DMSO was as controls. The representative dotplots were shown in left panel and the statistics were shown in right graph. The data are expressed as mean ± SEM of 4 biological replicates and representative of three independent experiments. *p < 0.05, ***p < 0.001, one-way ANOVA followed by Tukey's multiple comparisons test.

Given the data above showing the presence of high levels of IL-6 and IL-10 and their levels correlating with the abundance of CD14^+^HLA-DR^–/low^ MDSC in ascites from OC patients, we next defined whether AF-driven expansion of CD14^+^HLA-DR^–/low^ MDSC was due to the presence of IL-6 and/or IL-10 in the ascites from OC patients. To this end, we cultivated the PBMC freshly isolated from healthy donors with the AF from OC patients in the presence of neutralizing antibodies against IL-6 and/or IL-10 for 48 hours and then determined the frequency of CD14^+^HLA-DR^–/low^ MDSC by flow cytometry; as shown in Figure 5A, addition of either IL-6 or IL-10 neutralizing antibodies alone significantly attenuated the expansion of AF-driven CD14^+^HLA-DR^–/low^ MDSC with IL-6-specific neutralizing antibodies having a greater effect; furthermore, concomitant addition of both IL-6 and IL-10 neutralizing antibodies almost completely abrogated the expansive effect of AF on CD14^+^HLA-DR^–/low^ MDSC, indicating that the high levels of IL-6 and IL-10 in the ascites from OC patients were indispensable for the AF-driven expansion of CD14^+^HLA-DR^–/low^ MDSC.

As IL-6 and IL-10 engagement on their receptors leads to the activation of downstream STAT3 signaling pathway, we applied the Stattic, a small-molecule STAT3 inhibitor, to further define the role of IL-6 and IL-10 and their downstream signal in expansion of CD14^+^HLA-DR^–/low^ MDSC. We confirmed that addition of AF from OC patients into PBMC from healthy donors induced a rapid and durative activation of STAT3 signaling pathway where inclusion of Stattic as well as IL-6 and IL-10 neutralizing antibodies blocked STAT3 activation (Supplementary Figure 3). As expected, inhibition of STAT3 activation by Stattic abrogated the expansion of AF-driven CD14^+^HLA-DR^–/low^ MDSC similar to that done by neutralizing antibodies against IL-6 and IL-10 (Figure 5B), validating the critical role of both IL-6/IL-10 and their triggered downstream STAT3 signal in contributing to AF-driven expansion of CD14^+^HLA-DR^–/low^ MDSC.

To directly confirm the role of IL-6 and IL-10 in expansion of CD14^+^HLA-DR^–/low^ MDSC, we treated PBMC from healthy donors with recombinant human IL-6 (5 ng/ml) and IL-10 (0.5 ng/ml) at the concentrations similar to that found in the ascites from OC patients for 48 hours. As shown in Supplementary Figure 4, combined IL-6 and IL-10 treatment induced a significant expansion of CD14^+^HLA-DR^–/low^ MDSC which was dependent on STAT3 activation since STAT3 inhibitor Stattic abrogated this expansion; however, the extent of CD14^+^HLA-DR^–/low^ MDSC expansion induced by combined IL-6 and IL-10 did not reach the level achieved by the AF from OC patients, indicating that other unidentified mediators existing in the ascites from OC patients may play a role in the production of these MDSC.

### AF-induced CD14^+^HLA-DR^–/low^ MDSC executed the immunosuppressive activity via ARG1 and iNOS upregulated by AF-driven STAT3 activation

**Figure 6:**
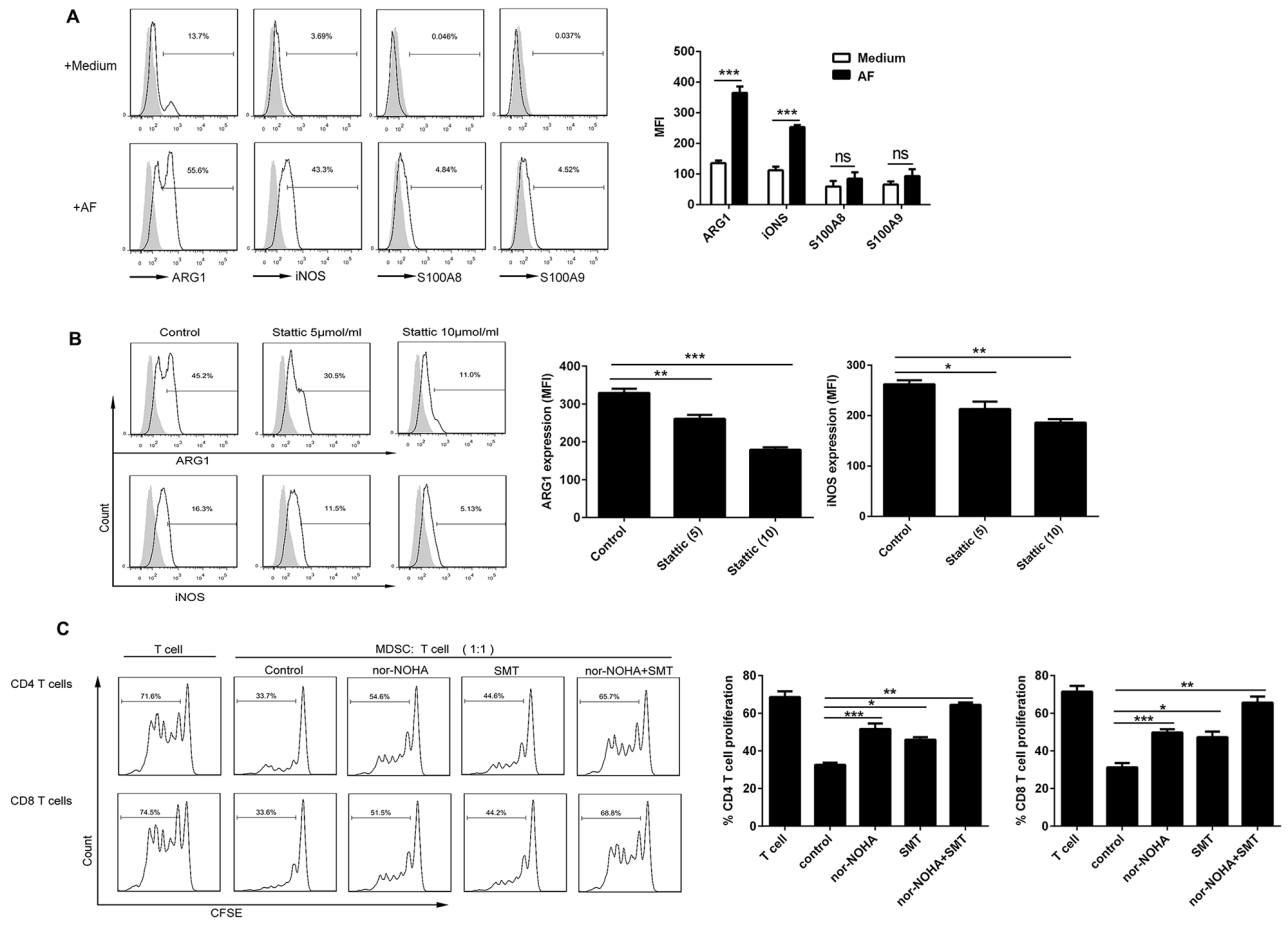
Both ARG1 and iNOS mediated the immunosuppressive activity of AF-induced CD14^+^HLA-DR^–/low^ MDSC PBMC from HD (n = 3) were treated with the AF (50% v/v) from OC patients (n=4) in the absence **(A)** or presence **(B)** of STAT3 inhibitor stattic for 48 hours and then analyzed for the expression of ARG1, iNOS and S100A8/A9 in CD14^+^HLA-DR^-/low^ MDSC by flow cytometry as well as **(C)** assayed for immune suppressive activity on autologous CD4^+^ and CD8^+^ T cell proliferation in the absence or presence of specific inhibitors for ARG1 (nor-NOHA; 20 μmol) and/or iNOS (SMT; 100 μmol). The representative dotplots were shown in left panel and the statistics were shown in right graph. The data are expressed as mean ± SEM of 4 biological replicates and representative of three independent experiments. *p < 0.05, **p < 0.01, ***p < 0.001, one-way ANOVA followed by Tukey's multiple comparisons test.

To delineate the suppressive mechanisms used by AF-induced CD14^+^HLA-DR^–/low^ MDSC, we analyzed the expression of ARG1, iNOS, S100A8 and S100A9, previously well-defined molecules critical for MDSC immunosuppressive function [7, 10], in these cells by flow cytometry. As shown in Figure 6A, the AF from ovarian patients predominantly upregulated the expression of ARG1 and iNOS in CD14^+^HLA-DR^–/low^ MDSC with slight increased expression of S100A8 and S100A9; we further confirmed that both ARG1 and iNOS expression were dependent on the STAT3 activation since STAT3 inhibition by Stattic abrogated their upregulation in AF-induced CD14^+^HLA-DR^–/low^ MDSC (Figure 6B).

We next examined whether ARG1 and/or iNOS play a pivotal role in the immunosuppressive activity of AF-induced CD14^+^HLA-DR^–/low^ MDSC. To do this, nor-NOHA and SMT, the small-molecule inhibitor specific for ARG1 and iNOS respectively, were used to determine the involvement of these two candidate molecules. As shown in Figure 6C, AF-induced CD14^+^HLA-DR^–/low^ MDSC suppressed the proliferation of autologous CD4^+^ and CD8^+^ T cells as described above, however, addition of either nor-NOHA or SMT into T cell/MDSC cocultures rescued T-cell proliferation with concomitant inclusion of nor-NOHA and SMT almost completely abrogating the immunosuppressive effect of those MDSC, pointing to the critical role of both ARG1 and iNOS in mediating the immunosuppressive activity of AF-induced CD14^+^HLA-DR^–/low^ MDSC.

## DISCUSSION

In this study, we analyzed the abundance, phenotype, function and/or generation of CD14^+^HLA-DR^–/low^ MDSC in the PB and accompanying ascites from OC patients. Briefly, increased frequency of CD14^+^HLA-DR^–/low^ MDSC was found in the PB of OC patients which is correlated with cancer stage, but not cancer subtype or histological grading; these MDSC express the typical surface markers of M-MDSC and are able to inhibit the autologous T-cell proliferation; we noted a predominant increase of these MDSC in the companying ascites from OC patients which presents a good correlation with the levels of IL-6 and IL-10 in those ascites; more importantly, the abundance of these MDSC in both PB and ascites as well as the levels of both IL-6 and IL-10 in the ascites is inversely associated with the RFS of OC patients. Furthermore, the AF from OC patients is able to readily induce the expansion of CD14^+^HLA-DR^–/low^ MDSC depending on IL-6/IL-10-STAT3 signaling pathway; these AF-induced CD14^+^HLA-DR^–/low^ MDSC suppress the T-cell proliferation and effector function by STAT3-controlled ARG1 and iNOS expression.

CD14^+^HLA-DR^−/low^ MDSC have been reported to contribute to tumor progression and/or therapy resistance, and increased abundance of these cells has been described in the PB and/or tumor tissues from hepatocellular carcinoma (HCC), melanoma, lung cancer, head and neck SCC (HNSCC), esophageal SCC and prostate cancer [15–23], and in several cancer types, has shown correlation with poor prognosis of cancer patients [15, 20, 22]. Herein, we observed a significantly elevated frequency of CD14^+^HLA-DR^−/low^ MDSC in both PB and ascites from OC patients with their abundance inversely correlating with RFS, which is consistent with recent finding showing inverse association between intratumoral CD45^-^CD33^+^ MDSC content and overall survival of OC patients [13]; these findings altogether indicate that CD33^+^ MDSC in multiple compartments of OC patients critically involve in the progression of OC with the potential of prognostic biomarker.

CD14^+^HLA-DR^−/low^ MDSC from OC patients express the typical surface markers of CD11b, CD33 and CD124. CD124 (IL-4Rα) is a subunit of the IL-4 and IL-13 receptor, both involved in MDSC activation, and has been postulated as a MDSC marker though its functional relevance for MDSC-mediated suppression is yet completely defined [24]. Interestingly, these cells expresses CD39 but not CD73, two ectonucleotidase molecules mediating conversion of immunostimulatory ATP into immunosuppressive adenosine with resulting adenosine acting to suppress Th1, Th2, CTL, and NK cells, among others [25]. A recent study showed that CD39 and CD73 are expressed by mouse MDSC and promote the expansion of and facilitate the suppressive activity of mouse MDSC [26]; therefore, it is possible that CD39 may play a role in mediating the suppressive activity of CD14^+^HLA-DR^−/low^ MDSC on T cells which should be evaluated in our future studies. Consistent with a previous study on melanoma patients [18], we detected variable level of CD80, CD83 and/or CD86 expression on CD14^+^HLA-DR^−/low^ MDSC in the PB and/or ascites from OC; however, their functional significance remains elusive. As these molecules play an important role in stimulating T cell responses, their expression on CD14^+^HLA-DR^−/low^ MDSC possibly could enable them to establish antigen-independent contact with T cells, allowing suppressive factors to act for a longer time and over shorter distances, which still warrant further investigation by using well-designed experiments [18]. Notably, we did not observe the expression of CD68, CD204 and CD206 on CD14^+^HLA-DR^−/low^ MDSC from OC, making them distinguishable from tumor-associated macrophages (TAM) with alternative polarization (M2) which has been well characterized and described to be correlated with RFS of OC patients [27]. As M-MDSC can differentiate into TAM within tumor tissues, it is very likely that same scenario occurs in the ascites and thus not surprising that they have the same clinically relevant prognostic significance [28].

It has been widely accepted that the accumulation of MDSC is driven by tumor-derived inflammatory cytokines and associated signaling pathway, notable IL-6 and its downstream STAT3 signal [7]. Consistent with this concept, we found that the abundance of CD14^+^HLA-DR^−/low^ MDSC is positively correlated with IL-6 concentration in the ascites, and incubation of PBMC with the ascites from OC patients induced the expansion of CD14^+^HLA-DR^−/low^ MDSC depending on IL-6 and their triggered STAT3 signal. As a major mediator of cancer-related inflammation, IL-6 is mainly derived from both cancer cells and cancer-associated immune cells, and it has been frequently reported to be increased in the sera, ascites and/or tumor tissue from OC patients correlating with the infiltration of myeloid cells and poor prognosis [29–32]. The underlying mechanisms by which IL-6 promotes the carcinogenesis and progression of OC are extremely complex and multifactorial [33]; our findings that IL-6 along with IL-10 and possibly other mediators promotes the expansion of CD14^+^HLA-DR^−/low^ MDSC provides an explanation for the increased presence of these immunosuppressive cells in the ascites and possible tumor tissues from OC patients thus adding another layer to the complex roles of IL-6. Our results are consistent with a recent study of esophageal squamous cell carcinoma (SCC) where IL-6 induced the accumulation of CD11b^+^CD14^+^HLA-DR^−^ MDSC by stimulating STAT3 signal and its level in plasma samples was correlated with tumor progression and poor prognosis [17]. Thus, targeting IL-6 would be a viable strategy to decrease/deplete the level of MDSC in cancer patients to promote the endogenous or induced antitumor immune responses considering that specific antibodies against IL-6 or their ligand IL-6R have been marketed, which needs to be tested in future clinical trials [34, 35].

We also found that IL-10 is involved in AF-driven CD14^+^HLA-DR^−/low^ MDSC expansion and its level in the ascites is negatively associated with RFS of OC patients, which is concordant with previous studies documenting increased IL-10 level in the sera and ascites from OC patients [36, 37]. The detailed mechanisms for IL-10 involvement of MDSC induction in this setting remain to be investigated though it is reasonably postulated that IL-10 synergistically promoted the expansion of MDSC by triggering STAT3 signal in combination with IL-6 [7, 38]. Notably, though neutralization of IL-6 and IL-10 completely abrogated the expansive effect of AF from OC patients for MDSC, combined recombinant IL-6 and IL-10 treatment did not induce the expansion of MDSC to the extent that achieved by the AF, indicating IL-6 and IL-10 play an essential but insufficient role and also pointing to a potentially complementary role played by other unappreciated mediators present in the ascites in the recruitment and accumulation of ascitic CD14^+^HLA-DR^−/low^ MDSC. In fact, previous studies have demonstrated that multiple cytokines such as IL-1β, TNF-α, VEGF, PEG2 etc. have involved in the accumulation of MDSC and their levels have been iteratively described elevated in the ascites from OC patients [7, 12, 37, 39–42]. Future studies are needed to clarify the independent or combinatorial roles of these cytokines in fostering MDSC generation in the ascites/tumor from OC patients and their clinical significance.

Previous studies have demonstrated the major role of STAT3 signaling pathway by mastering multiple genes critical for the expansion, differentiation and suppressive function of MDSC [7, 8], including ARG1, iNOS, S100A8/A9 etc; accordingly, we found that ascites-driven STAT3 signal concomitantly upregulates the expression of ARG1 and iNOS, two key enzymes for the immune suppressive activity of CD14^+^HLA-DR^−/low^ MDSC as evidenced by the reversal of MDSC's immunosuppressive activity in the presence of their specific inhibitors. It remains to be investigated whether other downstream STAT3-dependent target genes with the immunosuppressive effects (IDO, COX2, IL-10 etc.) was induced in this setting. As the target genes of STAT3, it is also very possible that ascites-derived IL-6/IL-10 induced their production in MDSC via STAT3 activation consequently constituting a positive feedback loop to perpetuating the expansion of MDSC, which needs more studies to clarify. In term of the key role as a regulator of the immunosuppressive properties of MDSC, STAT3 thus represents a candidate target for therapies aiming to reverse MDSC-mediated immunosuppression considering multiple small molecule drugs targeting STAT3 or JAK kinase have been developed over the past several years [43].

Our study has some limitations. First, the follow-up time for OC patients is relatively short (7-18 months after surgery) with a relatively small cohort which may generate a bias in interpreting the data about correlation analysis of RFS and MDSC level and limit the extrapolation of our findings; keeping a close follow-up of current patients and enrollment of more patients will contribute to address this question in future studies. Second, we did not perform simultaneous measurement of other key immune cell subsets such as T cells or B cells in both compartments; as recent studies showing that preoperative neutrophil-to-lymphocyte ratio can predict survival in OC patients [44, 45], it is very possible that the ratio of MDSC to T cells could more accurately predict the clinical outcome of OC patients, which will be checked in our future investigation.

In conclusion, we found the increased abundance of CD14^+^HLA-DR^−/low^ MDSC in the PB and ascites from OC patients that correlates with their poor prognosis; moreover, we show that ascites-derived IL-6 and IL-10 synergistically drive the expansion of these MDSC via STAT3 activation, thus providing a reasonable explanation for the predominantly elevated level of CD14^+^HLA-DR^−/low^ MDSC in the ascites from OC patients and complementing their origin other than CXCL12-CXCR4 mediated recruitment previously described [12]. As it has become clear that human MDSC play an important role in the immunosuppression of advanced cancer, various strategies systemically targeting MDSC have been widely developed via elimination, functional inactivation or maturation of MDSC though their efficacy remains to be improved [8]; with regard to OC, however, the efficacy could be optimized by using local delivery of MDSC-targeting agents such as STAT3 or IL-6 blockers into peritoneum thus enhancing the therapeutic efficacy of other OC modalities, which should be evaluated in future clinical trials.

## MATERIALS AND METHODS

### Patients

PBMC, sera and accompanying ascites were obtained from newly 31 diagnosed OC patients of four histological subtypes (serous, mucinous, endometrioid and mixed) in various stages (I-IV) at General Hospital of Chinese PLA, Beijing, China, from February 2015 to March 2016 and 31 age-matched healthy donors, after obtaining the written informed consent. These patients did not receive any preoperative chemoradiotherapy. The statistical and detailed demographics of those patients were shown in Supplementary Table 1 and 2. All patients underwent a primary surgical debulking procedure for clinical staging. Clinical courses were evaluated by RECIST criteria in patients with measurable disease or profiles of serum CA125 levels in patients without measurable lesions, according to the recommendations by the Gynecologic Cancer InterGroup (GCIG, http://www.gcig.igcs.org=CA-125.html).

### Cell isolation

PBMC were isolated from the PB of healthy donors and OC patients by density gradient centrifugation. In brief, blood was collected in EDTA-treated tubes, diluted 1/2 with RPMI 1640 medium, and carefully layered onto a density gradient Ficoll-Hypaque (GE Healthcare). After centrifugation, the band of PBMC was aspirated; PBMC were washed three times with ice-cold PBS containing 1% of human serum. Cell viability was checked by trypan blue dye exclusion.

For isolating immune cells from the ascites from OC patients, freshly ascites were collected aseptically and ascites cells were harvested by centrifugation from which immune cells were isolated using density gradient centrifugation as described above.

### Flow cytometric analysis

All monoclonal antibodies used in the study were purchased from Biolegend. For MDSC identification, we used PE-Cy7-anti-HLA-DR, FITC-anti-CD14 APC-anti-CD33 and/or APC-anti-CD45 antibodies. For phenotyping MDSC, we first gated MDSCs using anti-HLA-DR/CD14 antibodies and then costained with PE-anti-CD11b, CD13, CD15, CD39, CD73, CD80, CD83, CD86, CD124 (IL-4Rα), PD-1 (CD279), PD-L1 (CD274), and PD-L2 (CD273). For intracellular cytokine staining, cells were surface stained with anti-CD4/CD8 antibodies followed by permeabilization with the Cytofix/Cytoperm kit (BD Biosciences) and then staining with PE-anti-IL-2 or PE-anti-IFN-γ antibodies. Isotype-matched antibodies were used as controls. Data acquisition and analysis were performed using the flow cytometer (FC500 MPL, Beckman Coulter) and FlowJo software (Tree Star, Ashland, OR) respectively.

### Quantification of cytokines in the sera and ascites

The levels of IL-1β, IL-2, IL-4, IL-5, IL-6, IL-9, IL-10, IL-13, IL-17A, IL-22, IFN-γ and TNF-α cytokines were determined using the cytometric bead array system (BD eBioscience). Experiments were carried out following the manufacture's instruction manual. In brief, 50 μL of each vitreous body sample was incubated for 1 h with appropriate amounts of detection beads, which were specific for each investigated factor. Afterwards, samples were incubated for 2 h with detection reagent, which again was specific for each used detection bead. Samples were measured on a FACS Canto II and analyzed by FCAP array software.

### MDSC functional assay

CD14^+^HLA-DR^–/low^ MDSC and CD14^+^HLA-DR^+^ control cells in the PB and/or ascites from OC patients or in AF-treated PBMC from HD were purified by using MoFloTM XDP cell sorting system (Beckmam Coulter) with the purity of all isolated cell populations more than 95% (Supplementary Figure 1F). Purified MDSC and control cells were cocultured at the varying ratios with autologous PBMC labeled with 2 μM CFSE (Molecular Probe) according to the manufacturer's instructions in the presence of soluble anti-CD3 (2 μg/ml; Biolegend) and anti-CD28 (0.5 μg/ml; Biolegend) antibodies. After 72 hours, T-cell proliferation and IFN-γ production were assessed by CFSE dilution and intracellular cytokine staining by flow cytometry. In some instances, coculture was performed in the presence of specific inhibitors for ARG1 (nor-NOHA; Calbiochem) at 20 μmol and/or iNOS (SMT; Sigma) at 100 μmol.

### MDSC induction by AF

PBMC from healthy donors were cultured in the presence of varying concentrations (10, 30, 50% v/v) of AF from OC patients for 24, 48 or 72 hours, and resultant cells were analyzed for the frequency of or ARG1, iNOS, S100A8/A9 (Santa cruz) expression in MDSC by flow cytometry. In some instances, PBMC were cultured with the AF in the presence of neutralizing antibodies against IL-6 and/or IL-10 (Biolegend) or STAT3 inhibitor stattic (Selleck) with isotype antibody or DMSO as controls. In additional settings, PBMC from healthy donors were treated with recombinant human IL-6 and IL-10 (Peprotech) at indicated concentration with or without STAT3 inhibitor stattic for 48 hours.

### Western blotting analysis

Western blotting was done as we previously described [46] using the following primary antibodies: phosphorylated STAT3 (p-STAT3, Tyr705), total STAT3 and GAPDH from Cell Signaling Technology. HRP-conjugated goat anti-rabbit secondary antibodies (Cell Signaling Technology) were used for enhanced chemiluminescence of western blots.

### Statistical analysis

Statistical analysis was performed on GraphPad Prism 5.0 software (GraphPad Software, United States). Differences between groups were evaluated by two-tailed paired or unpaired Student's t test or one-way ANOVA followed by Tukey's multiple comparisons test; correlation between groups was evaluated by Pearson test. RFS was defined as the time period from date of surgery until disease recurrence due to OC and calculated using the Kaplan–Meier method. Survival differences between groups were assessed using Mantel–Cox log-rank test. P value <0.05 was considered statistically significant.

## SUPPLEMENTARY MATERIALS FIGURES AND TABLES




