# Supplementary material for: Ascites-derived IL-6 and IL-10 synergistically expand CD14+HLA-DR-/low myeloid-derived suppressor cells in ovarian cancer patients
Source: Oncotarget. 2017 Aug 10;8(44):76843–56. doi: 10.18632/oncotarget.20164 (PMC5652747; doi:10.18632/oncotarget.20164)
Supplement: Supplementary file 1 [file oncotarget-08-76843-s001.pdf]

Ascites-derived IL-6 and IL-10 synergistically expand $CD14^{+}HLA\text{-}DR^{-/low}$ myeloid-derived suppressor cells in ovarian cancer patients

SUPPLEMENTARY MATERIALS

Supplementary Table 1: Clinicopathological characteristics of the 31 OC patients

	N (%)
Age (years)	
Mean (SD)	55.77 (7.273)
FIGO stage	
Stage I	3 (9.7%)
Stage II	7 (22.6%)
Stage III	11 (35.5%)
Stage IV	10 (32.3)
Tumor type	
Serous	21 (67.7%)
Mucinous	5 (16.1%)
Endometrioid	3 (9.7%)
Mixed tumors	2 (6.5%)
Tumor grade	
Grade I	5 (16.1%)
Grade II	10 (32.3%)
Grade III	15 (48.4%)
Undifferentiated	1 (3.2%)
Lymph nodes	
Lymph nodes (+)	20 (64.5%)
Lymph nodes (-)	11 (35.5%)
Chemotherapy	
No chemotherapy	2(6.5)
Platinum-containing	7 (22.6%)
Platinum and taxane containing	21 (67.7%)
Other regimen	1 (3.2%)

FIGO=International Federation of Gynecology and Obstetrics

Supplementary Table 2: Detailed clinicopathological characteristics of the 31 OC patients

See Supplementary File 1

Supplementary Table 3: Correlation of $CD33^+CD14^+HLADR^-$ MDSC frequency with cytokine concentration in ascites

Parameter 1	Parameter 2	r	p
$CD33^+CD14^+HLADR^-$(MDSC)	IL-1beta	0.1603	0.2224
$CD33^+CD14^+HLADR^-$(MDSC)	IL-2	0.01812	0.6931
$CD33^+CD14^+HLADR^-$(MDSC)	IL-4	0.03399	0.5874
$CD33^+CD14^+HLADR^-$(MDSC)	IL-5	0.04514	0.5305
$CD33^+CD14^+HLADR^-$(MDSC)	IL-6	0.669	0.0021
$CD33^+CD14^+HLADR^-$(MDSC)	IL-9	0.01853	0.6898
$CD33^+CD14^+HLADR^-$(MDSC)	IL-10	0.7566	0.0005
$CD33^+CD14^+HLADR^-$(MDSC)	IL-13	0.008003	0.7937
$CD33^+CD14^+HLADR^-$(MDSC)	IL-17A	0.3014	0.0803
$CD33^+CD14^+HLADR^-$(MDSC)	IL-22	0.0437	0.5373
$CD33^+CD14^+HLADR^-$(MDSC)	IFN-γ	0.3047	0.0783
$CD33^+CD14^+HLADR^-$(MDSC)	TNF-α	0.01733	0.6996

*Pearsons r; significant values (p<0.05) indicated in red.

Supplementary Table 4: Correlations between cytokine concentrations in ascites*

Cytokine	IL-6	IL-10
IL-1beta	0.3877	0.9075
IL-2	0.1055	0.7733
IL-4	0.6264	0.4069
IL-5	0.4655	0.4276
IL-6	-	0.001
IL-9	0.6756	0.8984
IL-10	0.001	-
IL-13	0.9628	0.7216
IL-17A	0.1785	0.0789
IL-22	0.1851	0.6058
IFN-γ	0.1293	0.1818
TNF-α	0.7418	0.9458

*p-values of Pearsons r, n = 11; significant values (p<0.05) indicated in red.

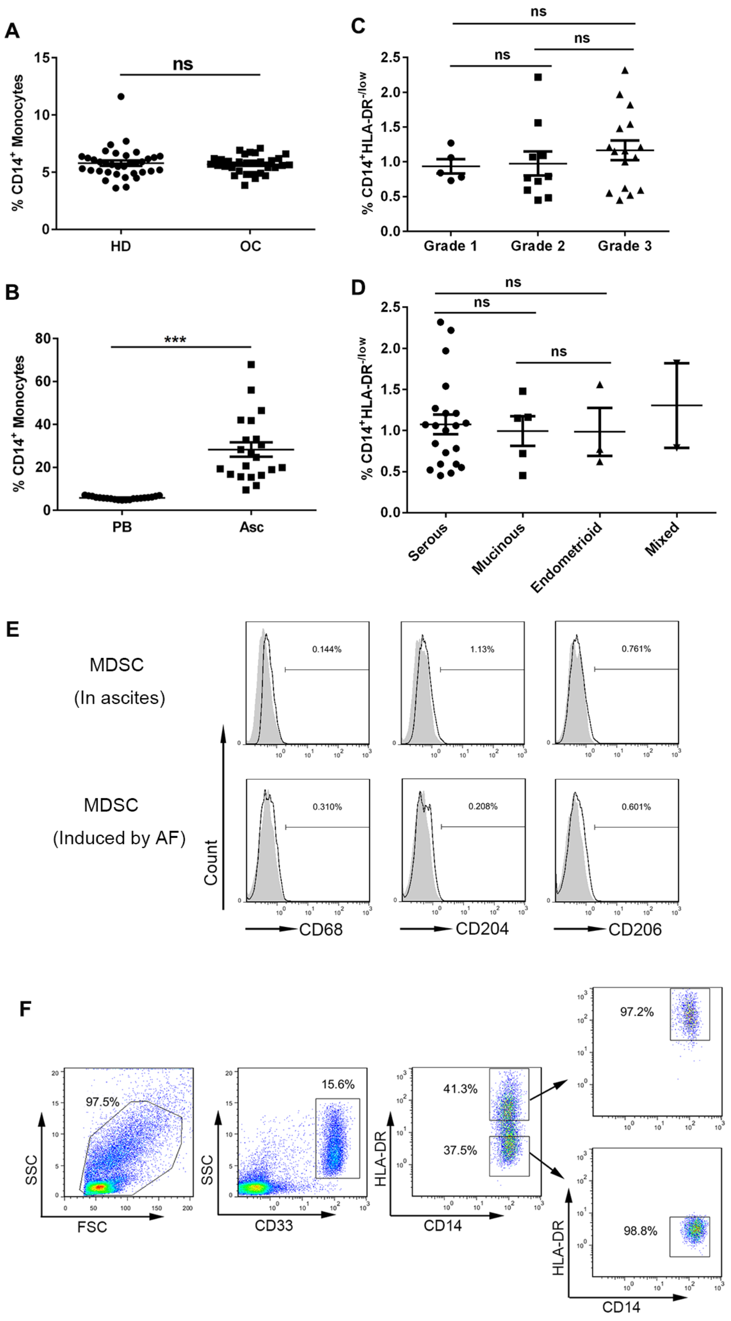

Supplementary Figure 1: (A) The abundance of circulating CD14$^+$ monocytes (relative to total CD45$^+$ leukocytes) in OC patients (n = 31) versus healthy donors (HD; n = 35). **(B)** The abundance of CD14$^+$ monocytes (relative to total CD45$^+$ leukocytes) in the PB versus accompanying ascites from OC patients (n = 21). **(C)** Relative abundance of circulating CD14$^+$HLA-DR$^{–/lo}$ MDSC in OC patients according to the histological grading. **(D)** Relative abundance of circulating CD14$^+$HLA-DR$^{–/lo}$ MDSC in OC patients according to the cancer subtypes. **(E)** Representative histograms of CD68, CD204 and CD206 TAM-associated markers on ascites-derived or AF-induced CD14$^+$HLA-DR$^{–/lo}$ MDSC with isotype controls as shaded areas. **(F)** Gating strategy to isolate CD14$^+$HLA-DR$^{–/lo}$ MDSC and control CD14$^+$HLA-DR$^+$ cells by flow cytometry.

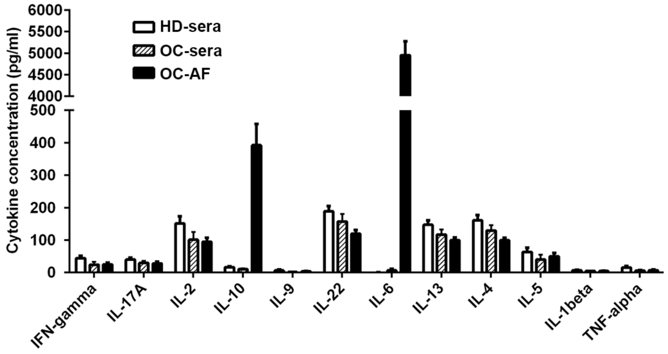

Supplementary Figure 2: The concentration of IL-1β, IL-2, IL-4, IL-5, IL-6, IL-9, IL-10, IL-13, IL-17A, IL-22, IFN-γ and TNF-α cytokines in the sera and/or ascites from HD (n=21) or OC patients (n=11) determined by the cytometric bead array (CBA).

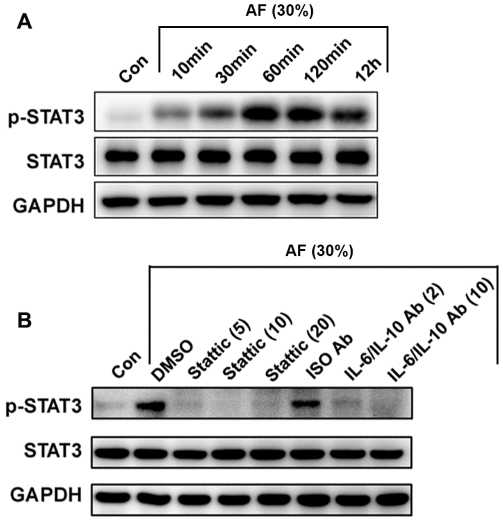

Supplementary Figure 3: (A) The phosphorylation status of STAT3 in the PBMC treated with the AF (30% v/v) from OC patients at the different time points. **(B)** The phosphorylation status of STAT3 in the PBMC treated with the AF (30% v/v) from OC patients in the presence of neutralizing antibodies against IL-6 and/or IL-10 or STAT3 inhibitor stattic with isotype antibody or DMSO as controls (30min after treatment).

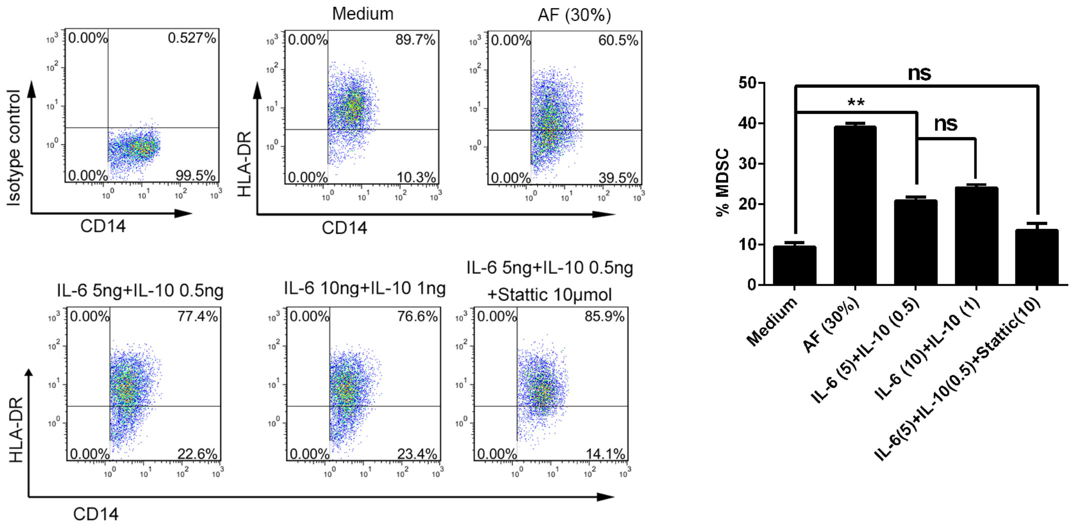

Supplementary Figure 4: PBMC from HD (n = 3) were treated with the AF (30% v/v) from OC patients (n = 3) or recombinant human IL-6 (5 or 10 ng/ml) and IL-10 (0.5 or 1 ng/ml) with or without STAT3 inhibitor stattic (10 µmol) for 48 hours and then analyzed for the abundance of CD14^{+}HLA-DR$^{-/low}$ MDSC by flow cytometry. The representative dotplots were shown in left panel and the statistics were shown in right graph. The data are expressed as mean ± SEM of 3 biological replicates and representative of two independent experiments. **p < 0.01, one-way ANOVA followed by Tukey's multiple comparisons test.
