# Supplementary material for: Ascites-derived IL-6 and IL-10 synergistically expand CD14+HLA-DR-/low myeloid-derived suppressor cells in ovarian cancer patients
Source: Oncotarget. 2017 Aug 10;8(44):76843–56. doi: 10.18632/oncotarget.20164 (PMC5652747; doi:10.18632/oncotarget.20164)
Supplement: Supplementary file 2 [file oncotarget-08-76843-s002.docx]

Supplementary Table 2: Detailed clinicopathological characteristics of the 31 OC patients

| Patient ID | Age (y) | Grade | Lymph  nodes | Stage (FIGO) | Tumor type | Date of surgery | Recurrence time (m) | Chemotherapy |
| --- | --- | --- | --- | --- | --- | --- | --- | --- |
| OC1 | 46 | G3 | + | Ⅳ | Serous | 2014.12.20 | 7 | Platinum/taxane |
| OC2 | 55 | G3 | + | Ⅳ | Serous | 2015.1.18 | 8 | Platinum/taxane |
| OC3 | 72 | G3 | + | Ⅲ | Serous | 2015.1.19 | 7 | Platinum/taxane |
| OC4 | 49 | G3 | + | Ⅳ | Serous | 2015.2.8 | 13 | Platinum/taxane |
| OC5 | 61 | G2 | + | Ⅳ | Serous | 2015.2.15 | 12 | Platinum/taxane |
| OC6 | 58 | G3 | + | Ⅲ | Serous | 2015.2.17 | 13 | Platinum/taxane |
| OC7 | 50 | G2 | + | Ⅳ | Serous | 2015.3.2 | 12 | Platinum/taxane |
| OC8 | 57 | G3 | + | Ⅲ | Serous | 2015.3.10 | 11 | Platinum/taxane |
| OC9 | 57 | G3 | + | Ⅲ | Serous | 2015.2.18 | Unknown | Platinum/taxane |
| OC10 | 61 | G3 | + | Ⅳ | Serous | 2015.3.14 | 12 | Platinum/taxane |
| OC11 | 68 | G2 | + | Ⅲ | Serous | 2015.3.14 | Unknown | Platinum/taxane |
| OC12 | 62 | G2 | + | Ⅳ | Serous | 2015.3.15 | Unknown | Platinum/taxane |
| OC13 | 67 | G2 | - | Ⅳ | Serous | 2015.3.15 | Unknown | Platinum/taxane |
| OC14 | 43 | G1 | - | Ⅰ | Serous | 2015.3.24 | No recurrence | No |
| OC15 | 47 | G1 | - | Ⅱ | Serous | 2015.3.29 | No recurrence | Platinum |
| OC16 | 54 | G3 | - | Ⅱ | Serous | 2015.4.10 | 17 | Platinum/taxane |
| OC17 | 58 | G2 | - | Ⅱ | Serous | 2015.3.11 | Unknown | Platinum/taxane |
| OC18 | 54 | G1 | - | Ⅰ | Serous | 2015.3.10 | No recurrence | Platinum/taxane |
| OC19 | 42 | G1 | - | Ⅱ | Serous | 2015.4.2 | 16 | No |
| OC20 | 49 | G1 | - | Ⅰ | Serous | 2015.1.12 | Unknown | Other regimen |
| OC21 | 51 | G1 | - | Ⅱ | Serous | 2015.1.19 | Unknown | Platinum |
| OC22 | 51 | G3 | + | Ⅲ | Mucinous | 2015.3.18 | 14 | Platinum/taxane |
| OC23 | 62 | G3 | + | Ⅳ | Mucinous | 2015.4.1 | 7 | Platinum |
| OC24 | 70 | G3 | + | Ⅲ | Mucinous | 2015.2.18 | Unknown | Platinum/taxane |
| OC25 | 59 | G3 | + | Ⅳ | Mucinous | 2015.2.22 | Unknown | Platinum/taxane |
| OC26 | 49 | G2 | - | Ⅱ | Mucinous | 2015.3.17 | No recurrence | Platinum |
| OC27 | 60 | G3 | + | Ⅲ | Mixed tumors | 2015.3.22 | 15 | Platinum/taxane |
| OC28 | 55 | G2 | + | Ⅲ | Mixed tumors | 2015.3.22 | Unknown | Platinum |
| OC29 | 54 | G3 | + | Ⅲ | Endometrioid | 2015.4.3 | 16 | Platinum |
| OC30 | 55 | G2 | + | Ⅲ | Endometrioid | 2015.3.24 | 12 | Platinum/taxane |
| OC31 | 53 | G2 | - | Ⅱ | Endometrioid | 2015.4.19 | No recurrence | Platinum |

FIGO=International Federation of Gynecology and Obstetrics
